# Secular trends and features of thalamic hemorrhages compared with other hypertensive intracerebral hemorrhages: an 18-year single-center retrospective assessment

**DOI:** 10.3389/fneur.2023.1205091

**Published:** 2023-08-15

**Authors:** Hiroyuki Katano, Yusuke Nishikawa, Mitsuru Uchida, Tomoyasu Yamanaka, Yuki Hayashi, Shigeki Yamada, Motoki Tanikawa, Kazuo Yamada, Mitsuhito Mase

**Affiliations:** ^1^Department of Neurosurgery, Graduate School of Medical Sciences, Nagoya City University, Nagoya, Japan; ^2^Department of Medical Informatics and Integrative Medicine, Graduate School of Medical Sciences, Nagoya City University, Nagoya, Japan; ^3^Nagoya City Rehabilitation Center Hospital, Nagoya, Japan

**Keywords:** hypertensive intracerebral hemorrhage, thalamic hemorrhage, putaminal hemorrhage, subcortical hemorrhage, secular change

## Abstract

**Introduction:**

Trends regarding the locations of hypertensive cerebral hemorrhages are unclear. To clarify hypertensive hemorrhage trends, we investigated intracerebral hemorrhages (ICHs) over an 18-year period, focusing on thalamic hemorrhages compared with other sites of hemorrhages.

**Methods:**

We reviewed the cases of patients hospitalized for hypertensive ICH in 2004–2021 at our hospital; 1,320 eligible patients were registered with a primary ICH/intraventricular hemorrhage. After exclusion criteria were applied, we retrospectively analyzed 1,026 hypertensive ICH cases.

**Results:**

The proportions of thalamic and subcortical hemorrhages increased over the 18-year period, whereas putaminal hemorrhage decreased. Multivariate logistic regression analyses revealed that for thalamic hemorrhage, ≥200 mmHg systolic blood pressure (*p* = 0.031), bleeding <15 mL (*p* = 0.001), and higher modified Rankin scale (mRS) score ≥ 4 at discharge (*p* = 0.006) were significant variables in the late period (2013–2021) versus the early period (2004–2012), whereas for putaminal hemorrhage, significant factors in the late period were triglyceride <150 mg/dL (*p* = 0.006) and mRS score ≥ 4 at discharge (*p* = 0.002). Among the features of the thalamic hemorrhages in the late period revealed by our group comparison with the putaminal and subcortical hemorrhages, the total and subcortical microbleeds were more notable in the thalamic hemorrhages than in the other two types of hemorrhage, whereas cerebellar microbleeds were more prominent when compared only with subcortical hemorrhages.

**Discussion:**

Our findings revealed an increasing trend for thalamic hypertensive hemorrhage and a decreasing trend for putaminal hemorrhage. The thalamic hemorrhage increase was observed in both young and older patients, regardless of gender. The main features of thalamic hemorrhage in the late period versus the early period were decrease in larger hemorrhage (≥15 mL) and an increase in cases with higher systolic blood pressure (at least partially involved a small number of untreated hypertensive patients who developed major bleeding). The total and subcortical microbleeds were more notable in the thalamic hemorrhages of the late period than in the putaminal and subcortical hemorrhages. These results may contribute to a better understanding of the recent trends of hypertensive ICHs and may help guide their appropriate treatments for this condition.

## 1. Introduction

Intracerebral hemorrhage (ICH) accounts for approximately 10–15% of all strokes and is associated with high morbidity and mortality (1–5). It has been reported that Asian populations (especially Japanese) experience ICH more frequently than Western populations (5, 6). Hypertension is the risk factor with the greatest influence on primary ICHs, and it is present in approx. 70% of patients who experience an ICH (7).

In a 1992 review concerning the location of ICHs, lobar hemorrhages accounted for 40–50%, lenticulate-capsular (putaminal) hemorrhages for 40%, followed by the cerebellum at 5–10% and the thalamus at 5% (8). The review’s authors noted that the proportion of lenticulate-capsular hemorrhages was greater when only patients with an ICH caused by hypertension were considered. A 2005 review stated that hypertensive hemorrhage in the basal ganglia accounted for 35–44% of ICH cases, whereas thalamic hemorrhages were 10–25% (9). In recent years, the percentage of thalamic hemorrhages in clinical practice appears to be increasing among hypertensive ICH patients. A population-based study conducted in Japan of the incidence of ICHs by their location revealed that the incidence of thalamic hemorrhage had increased particularly in individuals >70 years old, although in that study the ICHs had been diagnosed based solely on clinical manifestations (the Hisayama Study) (5). That study used data obtained up until 2001, and no apparent trends after that year have been reported. However, a recent cohort study using data from the Japan Stroke Data Bank reported secular changes in the functional outcomes of ischemic and hemorrhagic strokes (10).

Many investigators have used a national stroke database or community-based surveys regarding ICHs, including Japan (5, 10–12) and other countries (2, 3, 13–15). However, few reports have described secular changes regarding the proportion of hypertensive ICHs according to their locations, e.g., the thalamus, putamen, and other subcortical regions.

We retrospectively investigated secular trends in the cases of patients with hypertensive ICHs treated at our university hospital over an 18-year period (2004–2021), by the reported ICH sites. We sought to determine whether or not the proportion of thalamic hemorrhages increased over time among hypertensive ICH patients, even after the era of the Hisayama Study (5). We divided the 18-year period into first and second halves (2004–2012 and 2013–2021) and assessed the features of recent thalamic hemorrhages compared to the hemorrhages in the putamen and other sites.

## 2. Patients and methods

### 2.1. Patient population

We retrospectively analyzed the cases of the patients who were hospitalized with a hypertensive ICH by reviewing medical records from January 2004 (when our university hospital introduced electronic medical records) to December 2021. This 800-bed hospital is located in Nagoya, the third largest city in Japan (population 2.3 million), and it receives >1,000 new admissions in the neurological/neurosurgical departments annually. Initially, 1,320 eligible patients were registered with a primary ICH/intraventricular hemorrhage at the hospital, and after we applied the exclusion criteria a final total of 1,026 hypertensive ICH cases were retrospectively analyzed.

The exclusion criteria were as follows: patients with (1) a secondary ICH related to the following diseases and status: brain tumor, trauma, cerebral aneurysm, cerebral arteriovenous malformation, dural arteriovenous fistula, cavernous hemangioma, venous hemangioma, hemorrhagic cerebral infarction, sinus thrombosis, hyperperfusion syndrome, moyamoya disease, other underlying disease, or a status that tends to cause bleeding such as immune thrombocytopenia (ITP), leukemia, myelodysplastic syndromes (MDS), vasculitis, and the postpartum period; (2) an isolated intraventricular hemorrhage (IVH) (but including intraparenchymal hemorrhage with IVH by ventricular perforation); (3) hemorrhage in patients with systolic blood pressure (SBP) <130 mmHg without oral antihypertensives; (4) a subcortical hemorrhage with multiple superficial microbleeds (MBs) in the same lobe on MRI (before or at admission), in order to avoid typical cerebral amyloid angiopathy cases (16, 17).

**Figure 1:**
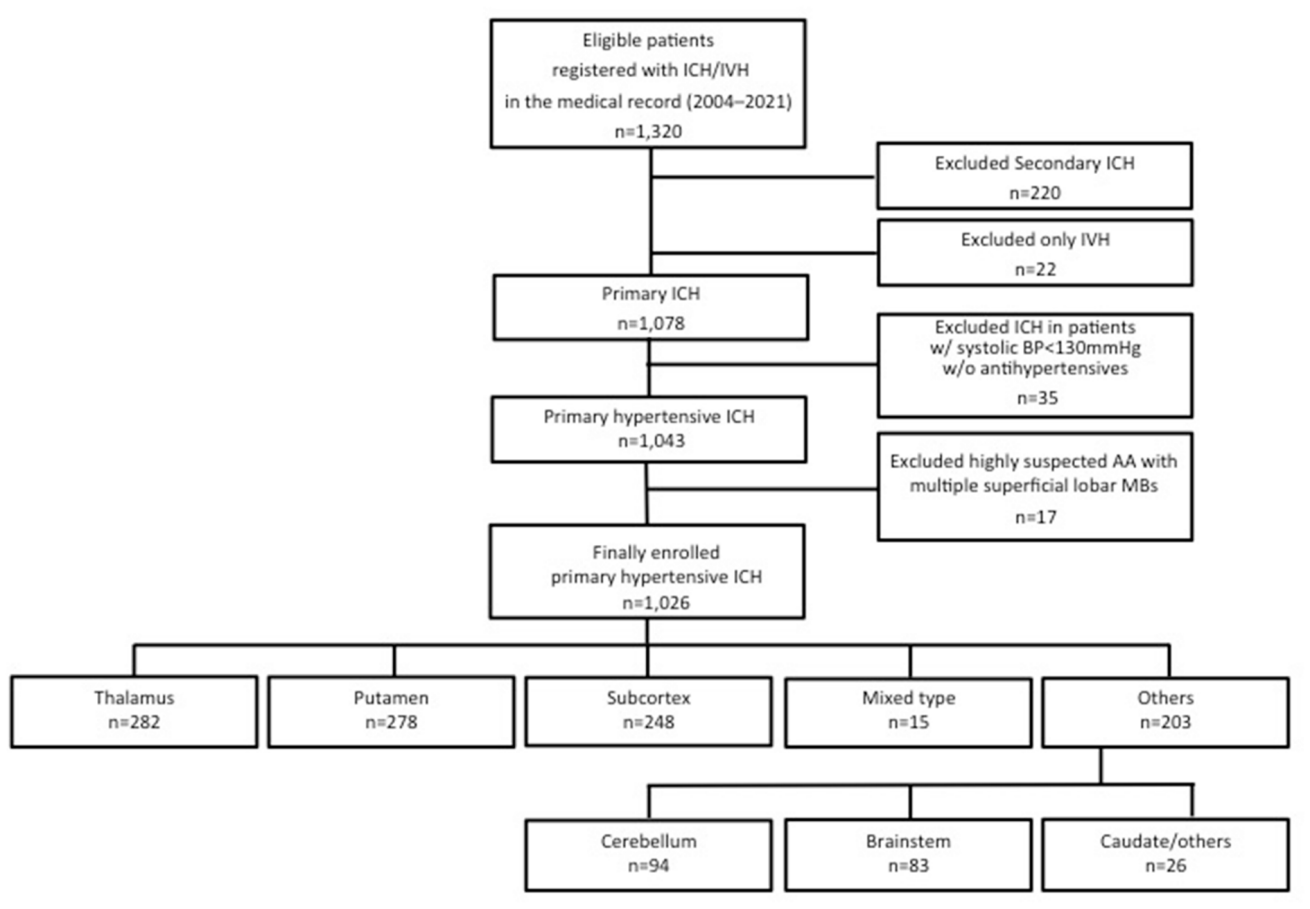
Patient inclusion and exclusion flow diagram. ICH, intracerebral hemorrhage; IVH, intraventricular hemorrhage; BP, blood pressure; AA, amyloid angiopathy; MB, microbleeds.

We classified the 1,026 cases into thalamic hemorrhage (*n* = 282), putaminal hemorrhage (*n* = 278), mixed-type of thalamic and putaminal hemorrhage (*n* = 15), subcortical hemorrhage (*n* = 248), cerebellar hemorrhage (*n* = 94), brainstem hemorrhage (*n* = 83), and caudate hemorrhage and others (*n* = 26) according to the predominant location of the hemorrhages on CT. Mixed-type hemorrhage was defined as a hemorrhage in which the large hematoma was located in both thalamic and putaminal areas and the origin of hemorrhage could not be determined radiographically; we treated mixed-type hemorrhage as different from thalamic and putaminal hemorrhages in the present analyses. Figure 1 provides the patient-enrollment flow diagram.

We examined temporal trends and characteristics of thalamic hemorrhage as well as the hemorrhages at other sites. We assessed the differences among the different ICHs during the 2004–2012 (early) and 2013–2021 (late) periods. We also compared the three main locations of hypertensive ICHs (i.e., the thalamus, putamen and subcortex) in each period to further elucidate the features of recent thalamic hemorrhages.

### 2.2. Imaging

The diagnosis of each hematoma as well as its location and volume was achieved with CT scans on admission by at least one neurosurgeon and one radiologist. A 16-detector-row CT scanner (IDT-16: Philips Medical Systems, Best, Netherlands) was usually used for the cases in the early period, and a 64-detector-row CT scanner (Somatom Definition: Siemens Medical Solutions, Forchheim, Germany) was used for the late period. Microbleeds were detected mainly with T2* images [TR/TE, 1,000/14 ms; section thickness, 5 mm; flip angle, 20°; acquisition matrix 512 × 512 ms; and field of view (FOV), 230 mm] or occasional susceptibility-weighted (SWI) images (TR/TE, 28/20 ms; section thickness, 2 mm; flip angle, 15°; bandwidth, 80 Hz/pixel; acquisition matrix 336 × 384 ms; and FOV, 201.25 × 230 mm) performed within 1 year before and occasionally after the ICH onset by MRI. A 1.5-T imaging system (Intera, Philip) was usually used in the early period, and a 3.0-T imaging system (Magnetom Avanto/Skyra, Siemens, Germany or Trillium Oval 3 T, Fujifilm, Tokyo) was used in the late period.

### 2.3. Investigated items for cases

The following factors were investigated: patient age, gender, body mass index (BMI), systolic blood pressure (SBP), diastolic blood pressure (DBP), blood sugar (BS) on admission, total cholesterol (TC), triglycerides (TG), low-density lipoprotein cholesterol (LDL-C), Glasgow Coma Scale (GCS) score on admission, length of hospital stay, the hematoma side and volume, the presence of an IVH, admission via the emergency room (ER), surgical method (craniotomy, stereotactic/endoscopic surgeries, drainage), medication before the onset [angiotensin-receptor blocker (ARB), calcium channel blocker (CCB) and other antihypertensive agents, aspirin and other antiplatelet agents, warfarin, direct oral anticoagulant (DOAC), or statin], comorbidities (diabetes, renal disease, heart disease, cerebral disease), habits [smoking history (past and current), current alcohol consumption], microbleed (MB) (posterior circulation, ipsilateral side, and multiple MB ≥3).

The detected specific sides/locations of MB were also investigated for the comparison between the different sites of hemorrhage within the same period. Multiple counts were allowed regarding the locations of MB. The percentage of 2nd bleeding and the Modified Rankin scale (mRS) score at discharge were also checked. The 2nd bleeding indicated a recurrent hypertensive intracerebral hemorrhage following a previous hypertensive hemorrhage, regardless of the current bleeding site. Regarding thalamic hemorrhages, specific hematoma locations were determined as anterior, lateral, medial, and posterior thalamus in accord with previous studies (18, 19).

Since 2013, the university hospital’s emergency department has been in full operation, all emergency patients were handed over to specialized departments after passing through the emergency department.

The study design was approved by our hospital’s Ethics Committee. The Helsinki Declaration and the ethical guidelines for life science/medical research involving human subjects issued by Japan’s Ministry of Health, Labour and Welfare (20) were strictly observed.

### 2.4. Statistical analyses

All analyses were performed with SPSS ver. 25.0 software (SAS, Cary, NC, United States). All data were analyzed using the Kolmogorov–Smirnov test for data distribution normality. All results are presented as mean ± SD values for normally distributed variables and median and 25th–75th percentiles for skewed variables, or the numbers and percentages. The Mann–Whitney U-test and χ^2^-test with Yates’ correction were applied for the comparison of continuous and categorical variables between two groups. A Kruskal-Wallis analysis followed by the Bonferroni method was used for comparisons among multiple groups. We conducted a two-way logistic regression analysis for the multivariate comparison. Correlations between variables were examined with Spearman’s correlation analysis. Probability (*p*)-values <0.05 were considered significant. Kappa (κ) statistics for the evaluation of inter-observer variability were used with 180 randomly selected cases for the radiological assessments (*κ* = 0.944).

## 3. Results

### 3.1. Secular trends and features of hypertensive ICH

**Figure 2:**
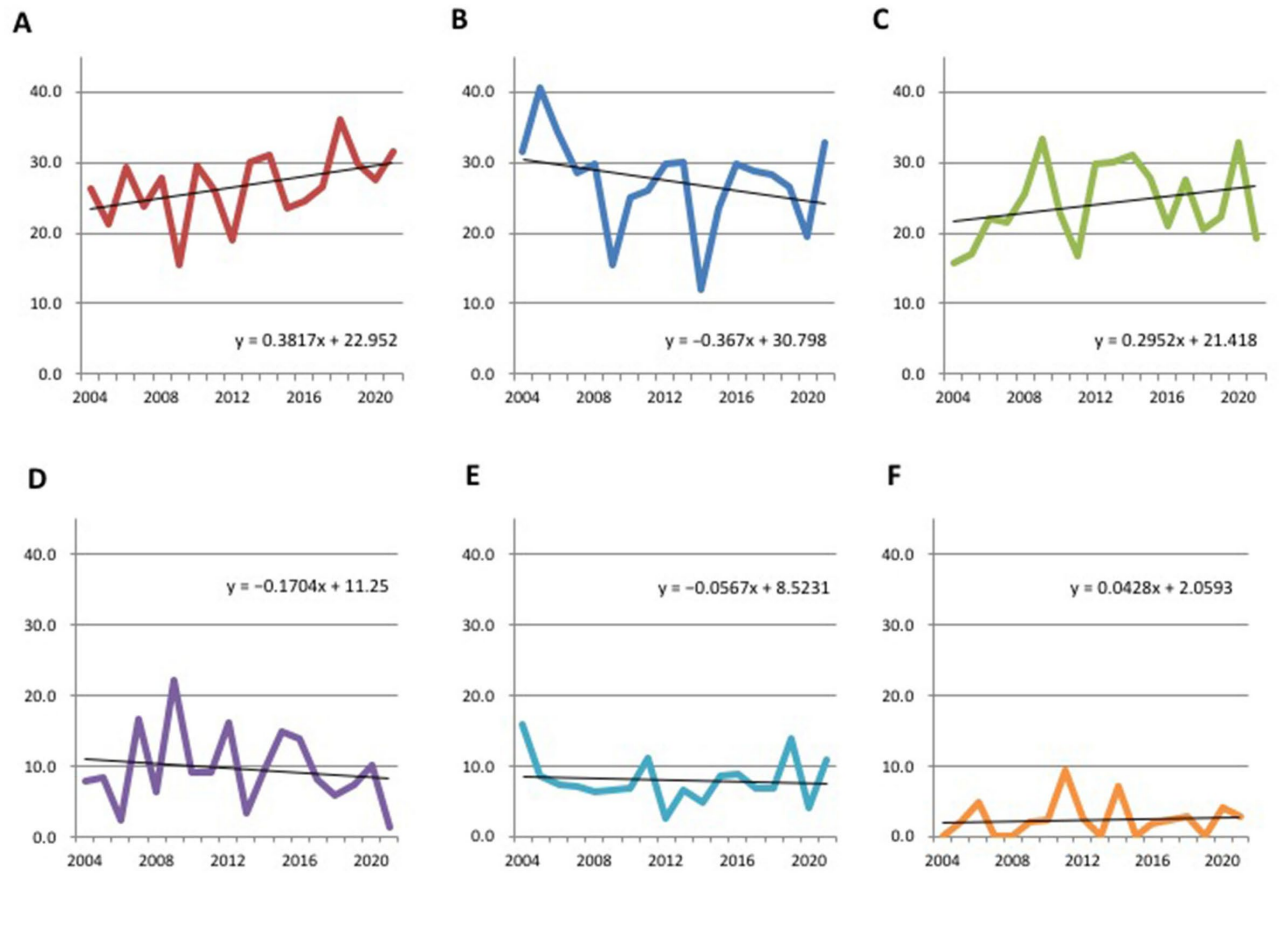
Secular changes in the ratio of each bleeding site of hypertensive intracerebral hemorrhage. **(A)** Thalamus. **(B)** Putamen. **(C)** Subcortex. **(D)** Cerebellum. **(E)** Brainstem. **(F)** Caudate/others.

**Figure 3:**
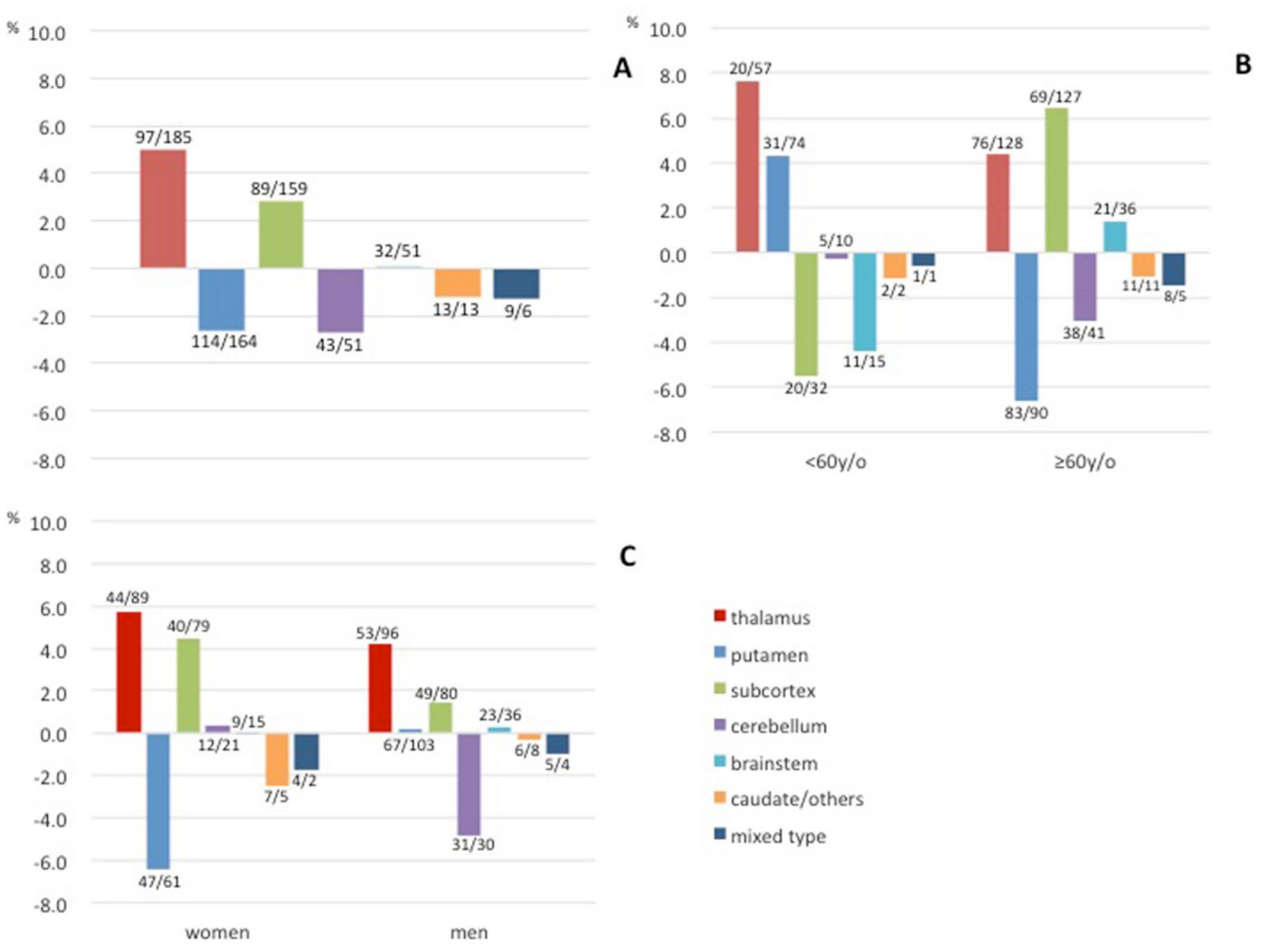
Differences in the proportions of each bleeding site of hypertensive intracerebral hemorrhage between the early (2004–2012) and late (2013–2021) periods. **(A)** Differences in all hemorrhages. **(B)** Differences according to age. **(C)** Differences according to gender. Early/late case counts are displayed near the bars.

The characteristics of all cases are provided in Table 1 and Figure 2 depicts the secular changes in the ratio of each bleeding site. Linear approximation showed that the incidence of thalamic hemorrhage tended to increase, as did that of subcortical hemorrhage, whereas putaminal hemorrhage and other hemorrhages showed a gradual decreasing trend or were almost flat (Figures 2A–F). Thalamic hemorrhage over time showed a moderate to strong significant correlation with a Spearman’s correlation coefficient of 0.453 (*p* = 0.03). The correlation coefficient of putaminal hemorrhage over time was 0.317, showing a moderate correlation, but *p* = 0.10. Subcortical hemorrhage (*r* = 0.243) and other hemorrhage sites over time were weakly correlated and not statistically significant. Comparing the cases in early period from 2004 to 2012 with those in the late period from 2013 to 2021, the proportion of thalamic hemorrhage among the total hemorrhages increased significantly by 5.01% (*p* = 0.047), whereas the proportion of subcortical hemorrhage increased by 2.67% and the proportion of putaminal and cerebellar hemorrhage decreased by 2.87 and 2.80%, respectively (Figure 3A and Supplementary Table S1). These decreases were not significant.

**Table 1:** Characteristics of all of the hemorrhages in the early and late periods.

Characteristic	All hemorrhages		*p*-value
	2004–2012 *n* = 395	2013–2021 *n* = 631	
Age, years	70.5 (60.2, 79.1)	68.7 (56.8, 78.6)	0.191
Women	163 (41.1%)	272 (43.2%)	0.490
BMI, kg/m^2^	21.1 (17.9, 24.4)	21.9 (19.3, 25.0)	0.588
BMI ≥23	103 (34.0%)	183 (31.0%)	0.358
SBP, mmHg	180.0 (157.0, 200.0)	176.0 (152.0, 200.0)	0.754
SBP ≥200 mmHg	123 (31.5%)	207 (33.1%)	0.594
DBP, mmHg	92.0 (80.0, 111.5)	98.0 (84.0, 118.0)	0.003*
DBP ≥100 mmHg	170 (43.5%)	302 (48.6%)	0.115
BS, mg/dL	131.0 (112.0, 163.0)	127.0 (108.0, 157.0)	0.032*
BS ≥150 mg/dL	141 (36.7%)	201 (32.8%)	0.433
TC, mg/dL	183.0 (163.0, 215.5)	190.0 (162.0, 217.0)	0.665
TC ≥230 mg/dL	45 (18.1%)	64 (15.9%)	0.634
TG, mg/dL	103.0 (79.0, 151.5)	92.0 (74.0, 130.0)	0.012*
TG ≥150, mg/dL	55 (24.4%)	64 (16.8%)	0.098
LDL-C, mg/dL	115.0 (87.0, 137.5)	110.0 (86.0, 137.0)	0.753
LDL-C ≥120 mg/dL	84 (41.8%)	150 (40.4%)	0.752
Hospital stay, days	21.0 (13.5, 36.5)	20.0 (14.0, 29.0)	0.069
GCS	14.0 (12.5, 15.0)	14.0 (11.0, 15.0)	0.584
GCS ≤9	101 (25.6%)	148 (23.6%)	0.467
Hemorrhage side, right	181 (47.1%)	277 (45.7%)	0.661
Hemorrhage volume, mL	9.5 (3.2, 25.0)	10.0 (4.0, 30.0)	0.346
Volume ≥15 mL	159 (40.1%)	251 (39.9%)	0.763
Hematoma with IVH	155 (39.0%)	210 (36.0%)	0.327
Hospitalization via ER	314 (79.1%)	586 (93.2%)	<0.001*
Surgery			
Craniotomy	33 (8.3%)	23 (3.7%)	0.001*
Stereotactic	15 (3.8%)	0 (0%)	<0.001*
Endoscopic	6 (1.5%)	14 (2.2%)	0.493
Drainage	21 (5.3%)	13 (2.1%)	0.005*
Medications			
ARB	64 (16.2%)	119 (18.9%)	0.257
CCB or other antihypertensive	72 (18.2%)	112 (17.8%)	0.888
Aspirin	40 (10.1%)	53 (8.4%)	0.361
Other antiplatelet	22 (5.6%)	30 (4.8%)	0.574
Warfarin	22 (5.6%)	29 (4.6%)	0.502
DOAC	0 (0%)	30 (4.8%)	<0.001*
Statin	27 (6.8%)	75 (11.9%)	0.008*
Diabetes	44 (11.1%)	58 (9.2%)	0.329
Any renal disease	23 (5.8%)	27 (4.3%)	0.275
Any heart disease	20 (5.1%)	65 (10.4%)	0.003*
Any cerebral disease	72 (18.2%)	93 (14.8%)	0.148
Smoking	44 (11.1%)	127 (20.8%)	<0.001*
Alcohol drinking	35 (8.9%)	104 (17.0%)	<0.001*
Microbleeds	122/224 (54.5%)	301/493 (61.1%)	<0.001*
Posterior MB	91/224 (40.6%)	213/493 (43.2%)	0.001*
Ipsilateral MB	68/224 (30.3%)	187/493 (37.9%)	<0.001*
>3 MB	53/224 (23.7%)	117/493 (23.7%)	0.517
2nd bleeding	25 (12.0%)	32 (13.5%)	0.127
mRS at discharge	3.0 (1.0, 4.0)	3.0 (2.0, 4.0)	0.151
mRS ≥4	177 (44.8%)	347 (56.2%)	<0.001*

The data are presented as median and 25th-75th percentiles or numbers and percentages (%). ARB, angiotensin-receptor blocker; BMI, body mass index; BS, blood sugar; CCB, calcium channel blocker; DBP, diastolic blood pressure; DOAC, direct oral anticoagulant; ER, emergency room; GCS, Glasgow Coma Scale; IVH, intraventricular hemorrhage; LDL-C, low-density lipoprotein-cholesterol; MB, microbleeds; mRS, Modified Rankin Scale; SBP, systolic blood pressure; TC, total cholesterol; TG, triglycerides; *p < 0.05.

Concerning the patients’ ages, only the thalamic hemorrhage increased in both the age group <60 years and the group of patients aged ≥60 years. The increase was greater in the younger age group (7.6% vs. 4.4%, Figure 3B). The rate of putaminal hemorrhage increased in the younger age group and decreased in the older age group, and the opposite was observed for subcortical hemorrhage. Regarding gender, the proportion of thalamic and subcortical hemorrhages increased in both women and men. A decrease in the proportion of putaminal hemorrhage in women and a decrease in the proportion of cerebellar hemorrhage in men were also observed (Figure 3C).

### 3.2. Features of recent hypertensive hemorrhages

To reveal specific features of recent hypertensive hemorrhages, we performed univariate analyses between the early and late periods for all hemorrhages as well as thalamic and putaminal hemorrhages (Tables 1–3). The variables for which significant differences were observed specific to thalamic hemorrhage were SBP ≥200 mmHg, short hospital stay, left-sided predominance, and hemorrhage <15 mL, whereas the significant variables specific to putaminal hemorrhage were younger age, TG <150 mg/dL, lower rate of 2nd bleeding, and higher mRS score at discharge. The multivariate logistic regression analysis for thalamic hemorrhage revealed that SBP ≥200 mmHg (*p* = 0.031), bleeding <15 mL (*p* = 0.001), and higher (≥4) mRS score at discharge (*p* = 0.006) were significant variables in the late period compared to the early period, whereas for putaminal hemorrhage, significant factors in the late period were TG <150 mg/dL (*p* = 0.006) and mRS score ≥ 4 at discharge (*p* = 0.002) (Table 4).

We performed multiple group comparisons by a Kruskal-Wallis analysis followed by the Bonferroni method between the thalamic, putaminal, and subcortical hemorrhages for each period (Tables 5, 6). Focusing on the significant difference between thalamic and putaminal hemorrhage only in the late period, the specific factors revealed were as follows: low BMI, SBP ≥200 mmHg, high GCS score, and high total MB. Similar results were obtained by the direct comparison of the thalamic and putaminal hemorrhages by a Mann–Whitney analysis (data not shown). When we focused on the significant differences between thalamic and subcortical hemorrhages only in the late period, we observed that higher GCS score, fewer craniotomy, smoking/drinking, and more MBs (cerebellar and subcortical MBs) were specific factors.

### 3.3. Systolic blood pressure and the thalamic hematoma volume

**Figure 4:**
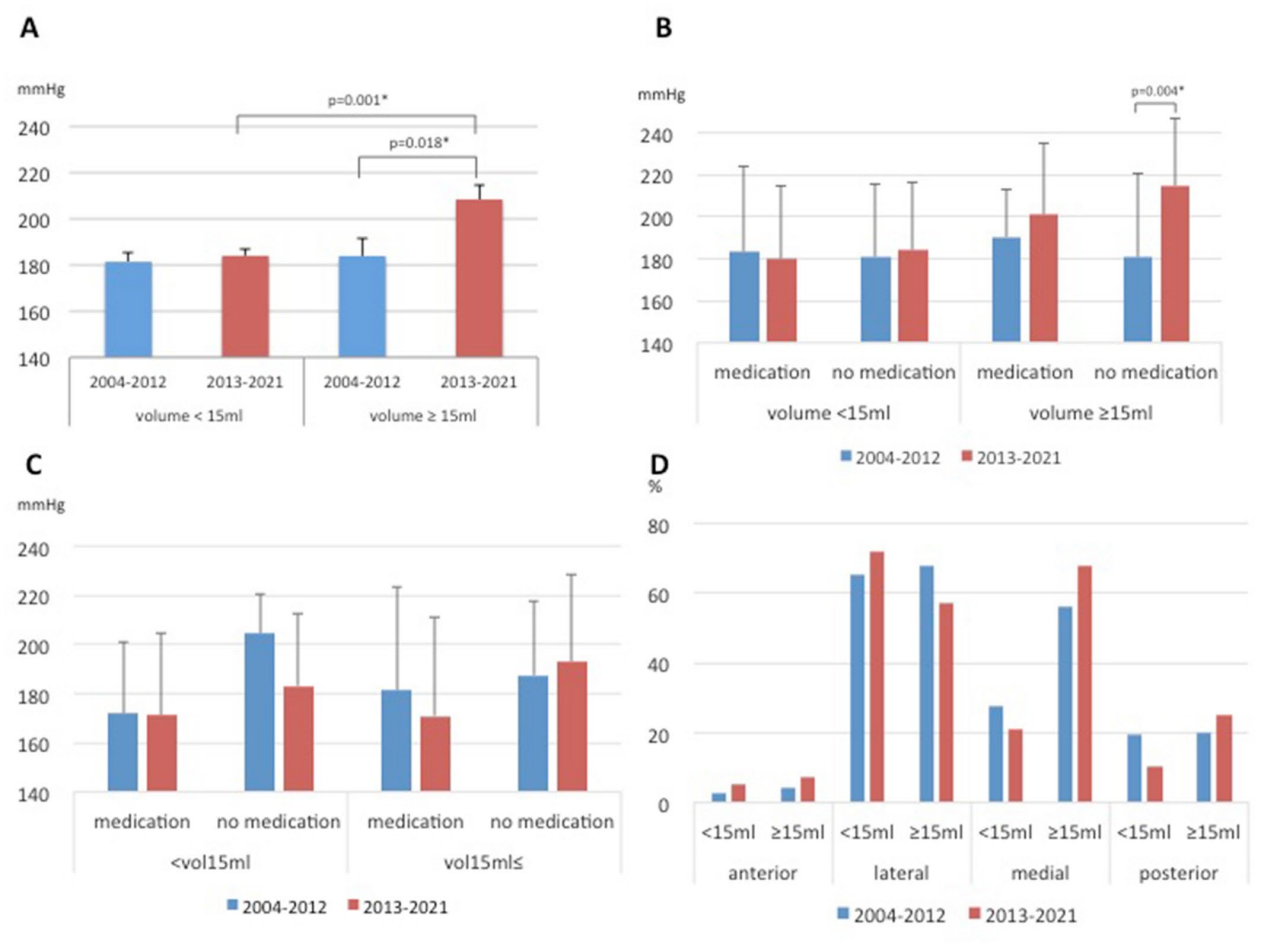
Differences in hypertensive intracerebral hemorrhages between the early and late periods. Systolic blood pressure (SBP) values according to the volume in the thalamic hemorrhages **(A)**, according to the volume in the thalamic hemorrhages and medication **(B)**, according to the volume in the putaminal hemorrhages and medication **(C)**. Proportions of specific hematoma locations in the thalamus of hypertensive thalamic hemorrhage **(D)**.

As a characteristic of the thalamic hemorrhages in the late period compared to the early period, the average SBP value was higher, although there were rather few cases with a large hemorrhage. The mean SBP was 208.4 ± 6.3 mmHg in the patients with a thalamic hemorrhage volume ≥ 15 mL in the late period, whereas the mean SBP was 184.0 ± 2.8 mmHg in the patients with a hemorrhagic volume < 15 mL (*p* = 0.001). In contrast, in the early period, there was no significant difference in the mean SBP values of the patients with thalamic hemorrhages between those with a volume ≥ 15 mL and those with a volume < 15 mL (183.9 ± 7.4 mmHg vs. 181.4 ± 4.3 mmHg, *p* = 0.409, Figure 4A). The SBP values of the patients with a thalamic hemorrhage under 15 mL showed no significant difference between the early and late periods regardless of medication use (*p* = 0.586 and *p* = 0.578). However, the mean SBP value among the patients with thalamic hemorrhages ≥15 mL in the late period was significantly higher than that in the early period, especially for the cases without medication (214.7 ± 32.0 vs. 180.7 ± 39.9 mmHg, *p* = 0.004, Figure 4B).

Concerning the patients with hemorrhages ≥15 mL, the percentage of patients without medication was higher in the late period than in the early period (data not shown). The mean SBP was generally higher in the late period in unmedicated patients, even those with putaminal and subcortical hemorrhages >15 mL, but no significant difference was seen as in the thalamic hemorrhages (Figure 4C and Supplementary Figure S1). Regarding the specific hematoma locations in the thalamus, the proportion of lateral origin was increased 7% in the late period, and this trend was observed in small hematomas (<15 mL) but without a significant difference (Figure 4D and Supplementary Figure S2). Although the mean volume of the thalamic hemorrhage of all cases showed no significant difference between the early and late periods (10.7 ± 14.6 vs. 10.2 ± 18.3 mL), whereas for the hemorrhages ≥15 mL, the mean volume was larger in the late period than in the early period (39.7 ± 35.2 vs. 28.7 ± 18.9 mL), although the difference was not significant (*p* = 0.573).

On the other hand, in the late period, 79.7% (94/118) of patients with blood pressure ≥ 200 mmHg and hematoma volume ≤ 15 mL were higher than 71.4% (25/35) in the early period (*p* < 0.001). In addition, 23.4% (15/64) of patients with blood pressure less than 200 mmHg and hematoma volume ≥ 15 mL were more common in early compared to 5.8% (4/69) in late (*p* < 0.001).

### 3.4. Concerning past history and antihypertensives

Out of 165 cases with a history of cerebral diseases, most were due to cerebral infarction, but unfortunately, it was not possible to identify the specific locations of all previous cerebral infarctions based on the medical records. Among other past brain disorders, there were two cases of brain aneurysm treatment, two cases of idiopathic normal pressure hydrocephalus, one case of cavernous angioma, and one case of chronic subdural hematoma that were evident from the recorded information. As for heart diseases, atrial fibrillation was observed in 58 cases among all hemorrhages. Other heart diseases included seven cases of angina pectoris, eight cases of old myocardial infarction (with four cases of percutaneous coronary intervention), five cases after valve replacement surgery, four cases of aortic aneurysm (dissection), and one case of chronic heart failure.

Among all hemorrhages, there were 282 cases (27.5%) of antihypertensive medication usage and 744 cases (72.5%) of non-usage. Specifically, for thalamic hemorrhage, the numbers of non-users in the early and late periods were 74 (76.3%) and 131 (70.8%) respectively. For putaminal hemorrhage, the numbers were 89 (69.5%) and 101 (71.6%) respectively. For subcortical hemorrhage, the numbers were 63 (70.8%) and 110 (69.2%) respectively.

For thalamic hemorrhage, 36.4% (early stage/late period = 30.4/38.9%) of patients taking antihypertensive drugs were taking two or more types of ARBs and Ca channel blockers/other antihypertensives (including combination drugs), putaminal hemorrhage decreased to 31.6% (35.9/27.5%) in the late period, and ARB increased from 40.0% in the previous period to 55.2% in the latter period as a single agent (for thalamic hemorrhage, ARB was 43.8% in the first period, and 51.5% in the latter period). As for subcortical hemorrhage, 33.3% (early/late = 30.8/34.7%) of the patients prescribed antihypertensive drugs were taking two or more types of antihypertensives (including combination drugs). ARB was 44.4% in the early period and 40.6% in the late period.

## 4. Discussion

The proportion of thalamic hemorrhages among the hypertensive intracranial hemorrhages increased in both women and men in the late period of 2013–2021 compared to the early period of 2004–2012. The reasons for these increases are unknown, but when we examined the differences between the two periods, we observed that many small bleedings (<15 mL) and SBP ≥200 mmHg on admission were the characteristic features of the late period. The patients who developed a ≥ 15-mL thalamic hemorrhage during the late period tended to have higher SBP at admission and thus might have not been treated for hypertension, and they had a greater amount of bleeding compared to the patients in the early period. On the other hand, as a matter of course, the number of patients with SBP ≥200 mmHg on admission and a hematoma volume of 15 mL or less was significantly higher in the late period than in the early period. This suggests that the size of the hematoma is not solely determined by the magnitude of blood pressure, but may be influenced by other factors such as individual differences in vascular fragility and anatomical variations.

### 4.1. Secular trend of thalamic hemorrhage

Wityk and Caplan reported that the proportion of thalamic hemorrhages among all hypertensive intracerebral hemorrhages was 5% (8). The rate of hypertensive thalamic hemorrhages was described as 10–25% in a review by Auer and Sutherland (9). A population-based cohort study (the Hisayama Study) revealed an increasing trend of thalamic hemorrhage up to 17.9% in a cohort from 1988 to 2001 (5), and the same group reported chronological changes in the incidence and mortality of cardiovascular diseases including ischemic strokes and myocardial infarction as well as whole ICH (11). A recent study using data from the Japanese Stroke Database reported that the proportion of thalamic hemorrhage was 28.1% based on an assessment of cumulative ICH cases from 1998 to 2018, but the cases were not strictly confined to hypertensive ICH and included hemorrhages from other causes (21). There have been few reports concerning secular trends of hypertensive hemorrhages focusing on the hemorrhage location since the above-mentioned Hisayama study (5). A recent study by Toyoda et al. focused on secular changes of the outcomes of ischemic and hemorrhagic stroke (10), but they did not refer to specific locations of the ICHs.

In our present analyses, the percentage of thalamic hypertensive hemorrhages in the late period was 29.3%, whereas that in the early period was 23.3%. We observed an increasing trend of thalamic hemorrhages as well as a decreasing trend of putaminal hemorrhages by linear approximation. This was in line with the results of the Hisayama study (5), and our present findings confirmed that this trend is continuing. Although thalamic hemorrhage showed a remarkable increasing secular trend in the Hisayama study (especially in people >70 years old), the results of our analyses demonstrated an increase in both young and older age groups, which is rather remarkable in people aged <60 years. In our cohort, even when considering 70 years as a cutoff point, the percentage of thalamic hemorrhage increased by 6.4% in the younger age group and increased by 4.2% in the older age group. On the other hand, putaminal hemorrhage increased by 1.4% in the younger age group and decreased by 7.3% in the older age group. The overall trends for thalamic and putaminal hemorrhages remained unchanged. These data seem to indicate a recent specific trend of hemorrhage compared to the previous studies.

### 4.2. Concerning selection for hypertensive ICH

Considering the research concerning hypertensive hemorrhage, it is important to note that a hemorrhage is actually caused by hypertension. In fact, it is difficult to strictly confine investigations to hypertensive ICH, and not a few studies have avoided classifying hypertensive ICHs but instead analyzed the cases as primary intracerebral hemorrhage or merely intracerebral hemorrhagic diseases including arteriovenous malformation or other hemorrhagic diseases such as cavernous angioma (2, 3, 5, 10–13, 21, 22). In the present study, we used several exclusion criteria and selected hypertensive hemorrhages as accurately as possible. The cases of patients with low blood pressure (<130 mmHg) at admission were excluded, and cases of ICH with comorbid diseases likely to cause a hemorrhage were also carefully excluded. It is quite difficult to exclude hemorrhages due to cerebral amyloid angiopathy (CAA). Because deep cerebral MBs are more commonly associated with hypertensive arteriopathy, and since strictly lobar (cortical–subcortical) cerebral MBs are likely to reflect CAA (16, 17), we excluded cases of clearly suspected CAA. However, it has been reported that solely based on the location of the hematoma, it is difficult to identify the difference in mortality rates between “possible” CAA-related hemorrhage and hypertensive hemorrhage (23). Additionally, Matsuyama et al. (24) suggested that mixed-type (strictly lobar and deep) MBs may be associated not only with hypertensive hemorrhage but also with CAA-related hemorrhage. Therefore, distinguishing hypertensive hemorrhage from CAA-related hemorrhage based solely on their locations may be still challenging.

### 4.3. The features of recent thalamic hemorrhages

Our analyses revealed that the recent thalamic hemorrhages had the characteristic of small hemorrhage (<15 mL). In the cases with smaller hemorrhages, the mean SBP values were not significantly different between the early and late periods. Moreover, hemorrhages in the lateral area [which are supplied mainly by the relatively large thalamogeniculate artery, which arises from the P2 segment of posterior cerebral artery (18, 19)] increased with rather small hemorrhages at the late stage. These findings might suggest a recent improvement in blood pressure control enabled by recent strong antihypertensives such as ARBs and calcium channel blockers or dietary control including low-salt diets (10). On the other hand, the number of patients with SBP ≥200 mmHg on admission was higher in the late period; this was at least partially accounted for by the significantly high SBP especially in the larger hemorrhages (≥15 mL), the number of which was small but whose amount was larger than that in the early period. Moreover, the higher SBP was identified in the patients who were not taking antihypertensive drugs, including those with untreated hypertension or self-discontinuance.

The short hospital stay detected as a significant variable in thalamic hemorrhage in the late period by the univariate analysis can be accounted for as a result of the implementation of requests for shorter hospitalization periods in recent years. Although there was no significant difference in all hemorrhages, from the perspective of medical economics and hospital management, the late period has seen a greater demand for shortened total hospital stay, especially in tertiary emergency hospitals such as university hospitals, compared to the early period. As a result, in the late period, compared to the early period, there may have been a greater tendency to transfer patients to secondary hospitals or specialized rehabilitation hospitals, at an earlier stage with higher mRS scores. This may have an impact on the higher mRS scores at discharge. Patients with thalamic hemorrhage with mRS4 at discharge showed the most significant difference, 14.4% of the total in the early stage and 26.5% in the late stage, which is considered to be the cause of the increase in the median and interquartile range of mRS and the percentage of patients with 4 or more.

Among the features of the thalamic hemorrhages in the late period revealed by our group comparison with the putaminal and subcortical hemorrhages, the total and subcortical MBs were more notable in the thalamic hemorrhages than in the other two types of hemorrhage, whereas cerebellar MBs were more prominent when compared only with subcortical hemorrhages. The lower number of MBs in the subcortical hemorrhages might have been affected by the exclusion criteria concerning MBs to exclude CAA.

Regarding smoking and alcohol consumption, univariate analysis showed a significant increase in thalamic hemorrhage as well as all hemorrhages in the late stage. When we compared thalamic hemorrhage, putaminal hemorrhage, and subcortical hemorrhage in the early and late stages respectively, and extracted items characteristic of thalamic hemorrhage, we found a significant difference in smoking and drinking only in the late stage. Therefore, smoking and alcohol consumption may somehow contribute to the secular increase in the rate of thalamic hemorrhage, but the mechanism has not been elucidated in this study. It is possible that the effects of smoking and alcohol consumption were more potent than those of subcortical or other hemorrhages, or possibly a combination of other factors like microbleeds as we discussed.

### 4.4. The trend and features of the putaminal hemorrhages

The significant factors in the univariate analysis concerning the putaminal hemorrhages only in the late period (not observed in all hemorrhage cases) were younger age, less recurrent cases, low TG, and worsening of discharge status (mRS score), and the latter two factors were also significant in the multivariate analysis. The reason for the lower triglyceride levels is not clear. There was no significant difference in triglyceride levels between the statin user group (*n* = 15) and the non-user group (*n* = 125) in the late period (*p* = 0.262). Therefore, statin use was not associated with lower triglyceride levels in the late period of putaminal hemorrhage. Additionally, there was no significant difference in LDL-C levels between the statin user group and the non-user group in both the early and late periods (*p* = 0.700 and *p* = 0.238, respectively), consistent with no difference in LDL-C between the both periods. This could be attributed to the low rate of statin use among the patients with putaminal hemorrhage in this study. In the analysis of all types of hemorrhage, there was no significant difference in triglyceride levels between the statin user group (*n* = 68) and the non-user group (*n* = 538) (*p* = 0.631). However, for LDL-C, there was a significant difference in the analysis of all types of hemorrhage, with a value of p of 0.032 between the statin user group (*n* = 66) and the non-user group (*n* = 507).

The elevated mRS score at discharge was considered to be influenced by the recent shortening of patients’ hospital stays, as discussed above for the thalamic hemorrhage. Patients with putaminal hemorrhage with a moderate severity level mRS4 at discharge showed the most significant difference, from 11.7% in the early period to 32.9% in the late period, which may have contributed to the increase in the average mRS and the percentage of patients with ≥4. There could be a possibility that the bleeding was severe and could have led to a poor prognosis due to factors such as direct involvement within internal capsule, as compared to the previous period. However, we have not conducted detailed investigations into these aspects at this time. The median and 75th percentile values are 9.5 and 20.3 mL, respectively, in the early period, and 10.7 and 27.8 mL in the late period, indicating that the bleeding amount in the late period has shifted to the larger side compared to the early period. Therefore, bleeding volume may also contribute to the poor outcome.

### 4.5. Differences between thalamic and putaminal hemorrhage

Sakamoto et al. (25) reported that putaminal hemorrhage occurs in younger patients (mainly those under 65 years old), while thalamic or subcortical (lobar) hemorrhage occurs in older patients. They also found a positive correlation between the small vessel disease burden score (white matter lesions, microbleeds, lacunar infarcts, and perivascular spaces enlargement) and thalamic hemorrhage, while putaminal hemorrhage showed an inverse correlation. They proposed that these differences could be explained by variations in the hemodynamics resulting from anatomical differences in the vasculature or differences in susceptibility to chronic hypertension and aging. According to the tsunami wave model by Saji et al., perforating arteries through the basal ganglia have a higher flow rate and larger amplitude of pulse pressure waves compared to superficial cortical arteries, making them more susceptible to significant pressure fluctuations (26). Additionally, deep ICH shows a stronger tendency for arterial sclerosis, leading to faster transmission of the pulse wave through the vascular walls (27). Tanaka et al. (28) have also noted that in cases of thalamic and putaminal hemorrhage, a prominent decrease in cerebral blood flow (CBF) occurs in the late stage of the former, highlighting differences in cerebral blood flow between thalamic and putaminal hemorrhages due to the involvement of diaschisis. While it is considered that some hemodynamic differences may arise between thalamic and putaminal hemorrhages, Sakamoto et al. suggest that putaminal hemorrhages are more likely to occur during the early stages of hypertension, whereas thalamic hemorrhages are frequently observed after a long period of hypertension, potentially involving sufficient small vessel disease pathology accumulated over time (25). They emphasize the possibility of involvement of not only anatomical changes but also the duration (age) and severity (blood pressure) of hypertension. Regarding age, in our univariate analysis, age was identified as a characteristic factor in putaminal hemorrhage in the late period, whereas no significant difference observed in thalamic hemorrhage (Tables 2, 3). Furthermore, in the comparison between early and late periods, thalamic hemorrhage increased in both the under 60 and over 60 age groups, while putaminal hemorrhage showed the highest decrease rate among all hemorrhage types in the over 60 age group (Figure 3B). Although this study is not a community study and does not examine age-specific changes in the population, Japan is experiencing a rapid increase in the elderly population, which could potentially contribute to the observed temporal increase in the proportion of thalamic hemorrhage and the temporal decrease in the proportion of putaminal hemorrhage. Regarding the small vessel disease burden score, this survey only considered MBs as a relevant factor, but particularly in the late stage, thalamic hemorrhage was significantly more prevalent than putaminal hemorrhage for all MBs, consistent with Sakamoto’s findings, suggesting it could be one of the contributing factors to the temporal increase. The distribution of MBs directly corresponded to the hemorrhage location, with 36.9% in thalamic hemorrhage and 13.0% in putaminal hemorrhage. Compared to putaminal hemorrhage, thalamic hemorrhage showed a higher probability of bleeding in the same location as the increasing MBs observed in the late stage. This finding is in line with the results reported by Sueda et al. (29), who investigated the positional relationship between recurrent ICH and prior MBs and described that the correspondence ratio was higher in the deep ICHs than the lobar ICHs. The increased occurrence of MBs in thalamic hemorrhage may contribute to explaining the increase over time.

**Table 2:** Characteristic of the thalamic hemorrhages in the early and late periods.

Characteristic	Thalamic hemorrhage		*p*-value
	2004–2012 *n* = 97	2013–2021 *n* = 185	
Age, years	70.6 ± 13.0	68.4 ± 16.8	0.305
Women	44 (45.4%)	89 (48.4%)	0.661
BMI, kg/m^2^	21.6 ± 4.4	21.5 ± 4.0	0.885
BMI ≥23	12 (15.4%)	38 (21.2%)	0.119
SBP, mmHg	182.6 ± 35.7	186.4 ± 33.5	0.376
SBP ≥200 mmHg	32 (33.7%)	97 (53.0%)	0.002*
DBP, mmHg	99.0 (90.0, 116.0)	99.5 (90.0, 114.8)	0.140
DBP≥100 mmHg	49 (52.1%)	89 (49.2%)	0.831
BS, mg/dL	124.5 (108.8, 155.3)	121.0 (104.3, 147.0)	0.050
BS ≥150 mg/dL	35 (37.2%)	47 (26.0%)	0.053
TC, mg/dL	194.0 (164.8, 221.0)	189.5 (165.5, 216.0)	0.289
TC ≥230 mg/dL	12 (17.4%)	19 (14.7%)	0.623
TG, mg/dL	107.0 (83.5, 149.0)	97.5 (75.3, 157.5)	0.405
TG ≥150 mg/dL	16 (25.8%)	32 (25.6%)	0.976
LDL-C, mg/dL	116.0 (87.8, 140.5)	110.0 (89.0, 136.8)	0.482
LDL-C ≥120 mg/dL	23 (42.6%)	48 (39.0%)	0.656
Hospital stay, days	27.0 (14.0, 36.8)	21.0 (14.0, 31.8)	0.045*
GCS	14.0 (12.3, 15.0)	14.0 (13.0, 15.0)	0.436
GCS ≤9	23 (24.0%)	31 (16.8%)	0.146
Hemorrhage side, right	55 (56.7%)	80 (43.2%)	0.032*
Hemorrhage location in thalamus			
Anterior	3 (2.5%)	10 (4.7%)	0.379
Lateral	64 (53.3%)	129 (60.3%)	0.520
Medial	34 (28.3%)	52 (24.3%)	0.229
Posterior	19 (15.8%)	23 (10.7%)	0.109
Hemorrhage volume, mL	5.0 (2.0, 14.9)	4.0 (2.0, 8.0)	0.430
Volume ≥15 mL	25 (25.8%)	28 (15.1%)	0.03^*^
Hematoma with IVH	54 (55.7%)	83 (47.4%)	0.193
Hospitalization via ER	74 (76.3%)	173 (93.5%)	<0.001*
Surgery			
Craniotomy	1 (1.0%)	1 (0.5%)	0.641
Stereotactic	2 (2.1%)	0 (0%)	0.050
Endoscopic	2 (2.1%)	0 (0%)	0.050
Drainage	8 (8.2%)	9 (4.9%)	0.257
Medications			
ARB	14 (14.4%)	39 (21.2%)	0.168
CCB or other antihypertensive	16 (16.5%)	35 (19.0%)	0.601
Aspirin	9 (9.3%)	15 (8.2%)	0.741
Other antiplatelet	5 (5.2%)	8 (4.3%)	0.745
Warfarin	7 (7.2%)	7 (3.8%)	0.211
DOAC	0 (0%)	12 (6.5%)	0.01*
Statin	7 (7.2%)	24 (13.0%)	0.138
Diabetes	14 (14.4%)	15 (8.2%)	0.100
Renal disease	4 (4.1%)	7 (3.8%)	0.896
Heart disease	4 (4.1%)	23 (12.5%)	0.024*
Cerebral disease	17 (17.5%)	25 (13.6%)	0.379
Smoking	7 (7.2%)	50 (27.6%)	<0.001*
Alcohol drinking	4 (4.1%)	38 (21.0%)	<0.001*
Microbleeds	32 (61.5%)	112 (74.2%)	0.084
Posterior MB	23 (44.2%)	95 (67.9%)	0.019*
Ipsilateral MB	19 (36.5%)	53 (56.4%)	0.900
>3 MB	15 (28.8%)	36 (40.9%)	0.473
2nd bleeding	4 (4.1%)	9 (4.9%)	0.778
mRS at discharge	3.0 (1.0, 4.0)	4.0 (2.0, 4.8)	0.287
mRS ≥4	42 (43.3%)	102 (55.4%)	0.053

The data are presented as mean ± SD values, median and 25th–75th percentiles or numbers and percentages (%); *p < 0.05. Abbreviations are explained in the footnotes of Table 1.

**Table 3:** Characteristics of the putaminal hemorrhages in the early and late periods.

Characteristic	Putaminal hemorrhage		*p*-value
	2004–2012 *n* = 114	2013–2021 *n* = 164	
Age, years	64.3 (58.1, 74.7)	62.5 (51.6, 77.3)	0.014*
Women	47 (41.2%)	61 (37.2%)	0.497
BMI, kg/m^2^	21.8 ± 4.7	22.5 ± 4.5	0.314
BMI ≥23	38 (33.3%)	57 (43.5%)	0.303
SBP, mmHg	181.4 ± 31.6	184.1 ± 33.1	0.595
SBP ≥200 mmHg	40 (35.1%)	47 (33.6%)	0.871
DBP, mmHg	91.5 (82.0, 110.5)	100.0 (88.0, 115.5)	0.009*
DBP ≥100 mmHg	49 (43.0%)	80 (57.6%)	0.055
BS, mg/dL	124.0 (112.0, 160.3)	131.0 (111.0, 158.0)	0.464
BS ≥150 mg/dL	35 (30.7%)	43 (30.7%)	0.833
TC, mg/dL	192.2 ± 35.7	193.9 ± 40.7	0.962
TC ≥230 mg/dL	14 (18.7%)	16 (17.4%)	0.932
TG, mg/dL	115.5 (83.8, 156.5)	90.0 (68.0, 120.0)	0.005*
TG ≥150 mg/dL	20 (28.6%)	11 (13.1%)	0.007*
LDL-C, mg/dL	116.3 ± 35.0	116.5 ± 33.1	0.800
LDL-C ≥120 mg/dL	30 (46.9%)	39 (48.8%)	0.840
Hospital stay, days	19.5 (13.8, 28.0)	18.0 (14.0, 26.5)	0.471
GCS	14.0 (12.0, 15.0)	14.0 (11.0, 15.0)	0.226
GCS ≤9	22 (19.3%)	35 (25.2%)	0.380
Hemorrhage side, right	45 (39.5%)	76 (46.3%)	0.256
Hemorrhage volume, mL	9.5 (4.2, 20.3)	10.7 (5.8, 27.8)	0.101
Volume ≥15 mL	49 (43.0%)	69 (49.3%)	0.451
Hematoma with IVH	24 (21.1%)	32 (19.5%)	0.707
Hospitalization via ER	91 (79.8%)	155 (94.5%)	<0.001*
Surgery			
Craniotomy	8 (7.0%)	6 (3.7%)	0.208
Stereotactic	11 (9.6%)	0 (0%)	<0.001*
Endoscopic	2 (1.8%)	6 (3.7%)	0.350
Drainage	1 (0.9%)	0 (0%)	0.235
Medications			
ARB	21 (18.4%)	31 (18.9%)	0.919
CCB or other antihypertensive	25 (21.9%)	26 (15.9%)	0.198
Aspirin	10 (8.8%)	11 (6.7%)	0.508
Other antiplatelet	6 (5.3%)	4 (2.4%)	0.214
Warfarin	8 (7.0%)	5 (3.0%)	0.123
DOAC	0 (0%)	9 (5.5%)	0.011*
Statin	8 (7.0%)	18 (11.0%)	0.265
Diabetes	18 (15.8%)	15 (9.1%)	0.092
Any renal disease	8 (7.0%)	9 (5.5%)	0.601
Any heart disease	4 (3.5%)	12 (7.3%)	0.180
Any cerebral disease	21 (18.4%)	22 (13.4%)	0.243
Smoking	15 (13.2%)	38 (23.2%)	0.033*
Alcohol drinking	14 (12.3%)	31 (18.9%)	0.131
Microbleeds	29 (46.8%)	65 (47.8%)	0.478
Posterior MB	22 (35.5%)	44 (34.1%)	0.820
Ipsilateral MB	13 (21.0%)	41 (31.8%)	0.063
>3 MB	10 (16.1%)	27 (20.9%)	0.289
2nd bleeding	7 (6.1%)	2 (1.2%)	0.030*
mRS at discharge	2.5 (1.0, 3.3)	4.0 (2.0, 5.0)	0.002*
mRS ≥4	39 (34.2%)	82 (49.4%)	<0.001*

The data are presented as mean ± SD values, median and 25th–75th percentiles or numbers and percentages (%). Abbreviations are explained in the footnotes of earlier tables; *p < 0.05.

**Table 4:** Multivariate logistic regression analysis predicting each hemorrhage in the late period.

	All hemorrhages		Thalamic hemorrhages		Putaminal hemorrhages	
	*p*-value	95%CI	*p*-value	95%CI	*p*-value	95%CI
Age <60 years	0.042*	1.017–2.598	0.234	0.693–4.489	0.093	0.885–4.882
Women	0.552	0.580–1.338	0.849	0.484–2.415	0.285	0.689–3.543
BMI ≥23	0.243	0.839–1.995	0.587	0.552–2.857	0.123	0.833–4.624
SBP ≥200 mmHg	0.293	0.482–1.246	0.031*	1.100–7.325	0.723	0.324–2.187
DBP ≥100 mmHg	0.810	0.682–1.631	0.289	0.279–1.463	0.189	0.739–4.606
BS ≥150 mg/dL	0.751	0.602–1.441	0.904	0.398–2.258	0.465	0.294–1.749
TC ≥230 mg/dL	0.979	0.532–1.848	0.943	0.270–3.376	0.303	0.555–6.647
TG ≥150 mg/dL	0.008*	0.303–0.837	0.583	0.298–1.976	0.006*	0.067–0.628
LDL-C ≥120 mg/dL	0.974	0.619–1.589	0.269	0.227–1.513	0.745	0.341–2.158
GCS ≤9	0.750	0.577–2.144	0.271	0.140–1.736	0.324	0.506–7.825
Vol ≥15 mL	0.01*	0.344–0.868	0.001*	0.043–0.461	0.070	0.134–1.080
mRS ≥4	<0.001*	1.694–4.352	0.006*	1.481–10.048	0.002*	1.718–12.013

BMI, body mass index; BS, blood sugar; DBP, diastolic blood pressure; GCS, Glasgow Coma Scale; LDL-C, low-density lipoprotein-cholesterol, mRS, Modified Rankin Scale, SBP, systolic blood pressure; TC, total cholesterol; TG, triglycerides; *p < 0.05.

**Table 5:** Group comparison of the thalamic, putaminal, and subcortical hemorrhages in the early period (2004–2012).

The early period (2004–2012) Characteristic	Kruskal-Wallis analysis *p*-value	Bonferroni method			
		Thalamus vs. putamen		Thalamus vs. subcortex	
		*p*-value	95%CI	*p*-value	95%CI
Age	0.061				
Women	0.800				
BMI	0.446				
BMI ≥23 kg/m^2^	0.538				
SBP	0.001*	1.000	−10.20–11.44	0.015*	2.02–25.14
SBP ≥200 mmHg	0.012*	1.000	−0.16–0.14	0.044*	0.00–0.33
DBP	0.003*	1.000	−4.72–9.73	0.006*	2.25–17.62
DBP ≥100 mmHg	0.041*	0.966	−0.10–0.23	0.036*	0.01–0.36
BS	0.138				
BS ≥150 mg/dL	0.383				
TC	0.880				
TC ≥230 mg/dL	0.808				
TG	0.265				
TG ≥150 mg/dL	0.911				
LDL-C	0.888				
LDL-C ≥120 mg/dL	0.861				
Hospital stay	0.084				
GCS	0.522				
GCS ≤9	0.570				
Hemorrhage side, right	0.029*	0.037*	0.01–0.34	0.114	−0.02–0.33
Hemorrhage volume	<0.001*	0.002*	−27.62 to −5.10	<0.001*	−33.90 to −9.96
Volume ≥15 mL	<0.001*	0.029*	−0.33 to −0.01	<0.001*	−0.48 to −0.15
Hematoma with IVH	<0.001*	<0.001*	0.19–0.50	0.004*	0.06–0.38
Hospitalization via ER	0.624				
Craniotomy	0.069				
Stereotactic	0.006*	0.027*	−0.15– −0.01	1.000	−0.06–0.08
Endoscopic	0.966				
Drainage	0.036*	0.035*	0.00–0.14	1.000	−0.05–0.10
ARB	0.705				
CCB or other antihypertensive	0.602				
Aspirin	0.662				
Other antiplatelet	0.669				
Warfarin	0.472				
DOAC	1.000				
Statin	0.967				
Diabetes	0.507				
Renal disease	0.530				
Heart disease	0.518				
Cerebral disease	0.769				
Smoking	0.360				
Alcohol drinking	0.104				
Microbleeds	0.201				
Pontine MB	0.010*	0.016*	0.02–0.29	0.016*	0.02–0.30
Ipsilateral cerebellar MB	0.816				
Contralateral cerebellar MB	0.059				
Ipsilateral thalamus	0.024*	0.021*	0.01–0.24	0.443	−0.05–0.19
Contralateral thalamus	0.002*	0.002*	0.05–0.28	0.511	−0.05–0.19
Ipsilateral basal ganglia	<0.001*	<0.001*	0.06–0.21	0.001*	0.04–0.19
Contralateral basal ganglia	0.003*	0.077	−0.01–0.22	0.002*	0.05–0.29
Ipsilateral subcortex	0.216				
Contralateral subcortex	0.046				
Posterior MB	0.003*	0.002*	0.07–0.42	0.031*	0.01–0.37
Ipsilateral MB	0.001*	0.001*	0.09–0.41	0.051	0.00–0.33
>3 MB	0.004*	0.003*	0.06–0.35	0.168	−0.03–0.27
2nd bleeding	<0.001*	0.001*	0.58–1.29	0.001*	0.51–1.22
mRS at discharge	0.590				
mRS ≥4	0.341				

ARB, angiotensin-receptor blocker; BMI, body mass index; BS, blood sugar; CCB, calcium channel blocker; DBP, diastolic blood pressure; DOAC, direct oral anticoagulant; ER, emergency room; GCS, Glasgow Coma Scale, IVH, intraventricular hemorrhage; LDL-C, low-density lipoprotein-cholesterol; MB, microbleeds; mRS, Modified Rankin Scale; SBP, systolic blood pressure; TC, total cholesterol; TG, triglycerides; *p < 0.05.

**Table 6:** Group comparison of the thalamic, putaminal, and subcortical hemorrhages in the late period (2013–2021).

The late period (2013–2021) Characteristics	Kruskal-Wallis analysis *p*-value	Bonferroni method			
		Thalamus vs. putamen		Thalamus vs. subcortex	
		*p*-value	95%CI	*p*-value	95%CI
Age	0.016*	1.000	−2.87–6.03	0.399	−6.92–1.59
Women	0.046*	0.122	−0.24–0.02	1.000	−0.11–0.14
BMI	0.001*	0.157	−2.08–0.22	0.461	−0.48–1.87
BMI ≥23 kg/m^2^	<0.001*	<0.001*	−0.35 to −0.12	1.000	−0.15–0.09
SBP	<0.001*	1.000	−6.09–10.62	0.000	13.36–30.21
SBP ≥200 mmHg	<0.001*	<0.001*	0.07–0.30	<0.001*	0.31–0.54
DBP	<0.001*	1.000	−7.21–4.36	<0.001*	5.09–16.74
DBP ≥100 mmHg	0.008*	0.715	−0.19–0.07	0.137	−0.02–0.24
BS	0.077				
BS ≥150 mg/dL	0.210				
TC	0.664				
TC ≥230 mg/dL	0.784				
TG	0.242				
TG ≥150 mg/dL	0.005*	0.025*	0.01–0.25	0.011*	0.03–0.28
LDL-C	0.621				
LDL-C ≥120 mg/dL	0.381				
Hospital stay	0.177				
GCS	0.002*	0.038*	0.04–1.80	0.001*	0.41–2.18
GCS ≤9	0.013*	0.339	−0.18–0.04	0.010*	−0.24 to −0.03
Hemorrhage side, right	0.715				
Hemorrhage volume	<0.001*	<0.001*	−27.68 to −11.11	<0.001*	−38.94 to −22.33
Volume ≥15 mL	<0.001*	<0.001*	−0.44 to −0.21	<0.001*	−0.67 to −0.45
Hematoma with IVH	<0.001*	<0.001*	0.12–0.37	0.019*	0.02–0.27
Hospitalization via ER	0.250				
Craniotomy	0.003*	0.369	−0.08–0.02	0.002*	−0.12 to −0.02
Stereotactic	1.000				
Endoscopic	0.038*	0.057	−0.07–0.00	0.137	−0.07–0.01
Drainage	<0.001*	0.002*	0.01–0.08	0.002*	0.01–0.08
ARB	0.694				
CCB or other antihypertensive	0.369				
Aspirin	0.322				
Other antiplatelet	0.153				
Warfarin	0.814				
DOAC	0.364				
Statin	0.839				
Diabetes	0.933				
Renal disease	0.388				
Heart disease	0.277				
Cerebral disease	0.802				
Smoking	0.001*	1.000	−0.06–0.15	0.001*	0.06–0.27
Alcohol drinking	0.024*	1.000	−0.08–0.11	0.029*	0.01–0.21
Microbleeds	<0.001*	<0.001*	0.11–0.38	0.001*	0.07–0.35
Pontine MB	<0.001*	0.009*	0.02–0.23	<0.001*	0.20–0.40
Ipsilateral cerebellar MB	0.008*	1.000	−0.06–0.13	0.008*	0.02–0.21
Contralateral cerebellar MB	<0.001*	0.083	−0.01–0.19	<0.001*	0.09–0.28
Ipsilateral thalamus	<0.001*	0.001*	0.05–0.26	<0.001*	0.13–0.33
Contralateral thalamus	<0.001*	<0.001*	0.09–0.30	<0.001*	0.20–0.40
Ipsilateral basal ganglia	0.001*	0.949	−0.04–0.11	<0.001*	0.04–0.18
Contralateral basal ganglia	0.001*	1.000	−0.08–0.08	0.002*	0.03–0.19
Ipsilateral subcortex	<0.001*	0.002*	0.05–0.29	<0.001*	0.09–0.32
Contralateral subcortex	<0.001*	<0.001*	0.08–0.32	<0.001*	0.07–0.31
Posterior MB	<0.001*	<0.001*	0.14–0.40	<0.001*	0.28–0.53
Ipsilateral MB	0.840				
>3 MB	0.562				
2nd bleeding	0.002*	0.014*	0.04–0.45	0.001*	0.08–0.39
mRS at discharge	0.789				
mRS ≥4	0.711				

ARB, angiotensin-receptor blocker; BMI, body mass index; BS, blood sugar; CCB, calcium channel blocker; DBP, diastolic blood pressure; DOAC, direct oral anticoagulant; ER, emergency room; GCS, Glasgow Coma Scale; IVH, intraventricular hemorrhage; LDL-C, low-density lipoprotein-cholesterol; MB, microbleeding; mRS, Modified Rankin Scale; SBP, systolic blood pressure; TC, total cholesterol; TG, triglycerides; *p < 0.05.

### 4.6. Study limitations

The major limitations of this study are its retrospective nature and the relatively small number of cases from a single institution. However, in a large-scale study in which many facilities participate, it is sometimes difficult to set detailed parameters at the time of data collection. We took advantage of single-center data analyses, which allowed us to focus on hypertensive factors and to perform more detailed analyses limited to specific hemorrhage sites. Second, although we performed MRI T2*/SWI in 71.9% (200/278) of all cases with subcortical hemorrhage and cases with clearly suspected amyloid angiopathy were excluded, the possibility that the subcortical hemorrhages in the present cohort included bleeding due to causes other than hypertension cannot be denied. Recently, the Boston criteria version 2.0 for diagnosing CAA has been introduced (30). However, our dataset for this study did not include evaluations of non-hemorrhagic lesions, which are incorporated in the Boston criteria version 2.0, making its application unfeasible in our cases. Nevertheless, even if the Boston criteria version 2.0 were used for CAA diagnosis, it is important to note that the false-positive rate is not 0% (ranging from 10.5 to 18.5% in the derivation and temporal validation cohort, and 5.0% even in the geographical validation cohort). As our study primarily focused on hypertensive intracerebral hemorrhage, we should prioritize comprehensive inclusion of hypertensive intracerebral hemorrhage cases, even if it meant potential false negatives for CAA. Third, as radiographic equipment and technology have naturally advanced with the times, we did not conduct a comparison of specific sites of MB between the early and late periods, and we indirectly compared the specific differences between each hemorrhagic location in each study period. This might also have affected the differential diagnosis of hypertensive subcortical hemorrhage and CAA. Forth, as described in the Result, out of 165 cases with a history of cerebral diseases, most were due to cerebral infarction, but unfortunately, it was not possible to identify the specific locations of all previous cerebral infarctions based on the medical records. Finally, the data from the years 2020–2021 might have been affected by the effects of the COVID-19 pandemic on the admission and treatment for hypertensive ICH.

## 5. Conclusion

Our findings revealed an increasing secular trend of thalamic hypertensive hemorrhages as well as a decreasing secular trend of putaminal hemorrhages. The increasing trend in thalamic hemorrhages was observed in in both young and older patients regardless of gender. The main features of thalamic hemorrhages in the late period (2013–2021) compared to the early period (2004–2012) were a decrease in larger hemorrhages (≥15 mL) and an increase in cases with higher SBP. The latter result could be at least partially attributed to the small number of untreated hypertensive patients who developed major bleeding. The total and subcortical MBs were more notable in the thalamic hemorrhages of the late period than in the putaminal and subcortical hemorrhages. Together the present results may contribute to a better understanding of the recent trend of hypertensive intracerebral hemorrhages and may help guide appropriate treatments for this condition.

## Data availability statement

The original contributions presented in the study are included in the article/Supplementary material, further inquiries can be directed to the corresponding author.

## Ethics statement

The studies involving humans were approved by the Local Ethics Committee of the Nagoya City University Graduate School of Medical Sciences (IRB number: 60-20-0148). The studies were conducted in accordance with the local legislation and institutional requirements. The ethics committee/institutional review board waived the requirement of written informed consent for participation from the participants or the participants' legal guardians/next of kin because written informed consent was not required for this study in accordance with national legislation and the institutional requirements.

## Author contributions

HK led the initiative and wrote the original draft. YN, MU, TY, and YH extracted the data and created the database. HK, SY, and MT were involved in data analysis and revised the drafted document. KY and MM were involved in supervision. All authors were involved in the conceptualization, review, editing and approved the final submitted version.

## Conflict of interest

The authors declare that the research was conducted in the absence of any commercial or financial relationships that could be construed as a potential conflict of interest.

## Publisher’s note

All claims expressed in this article are solely those of the authors and do not necessarily represent those of their affiliated organizations, or those of the publisher, the editors and the reviewers. Any product that may be evaluated in this article, or claim that may be made by its manufacturer, is not guaranteed or endorsed by the publisher.
