# Supplementary material for: Secular trends and features of thalamic hemorrhages compared with other hypertensive intracerebral hemorrhages: an 18-year single-center retrospective assessment
Source: Front Neurol. 2023 Aug 15;14:1205091. doi: 10.3389/fneur.2023.1205091 (PMC10464616; doi:10.3389/fneur.2023.1205091)
Supplement: Supplementary file 1 [file Image_1.pdf]

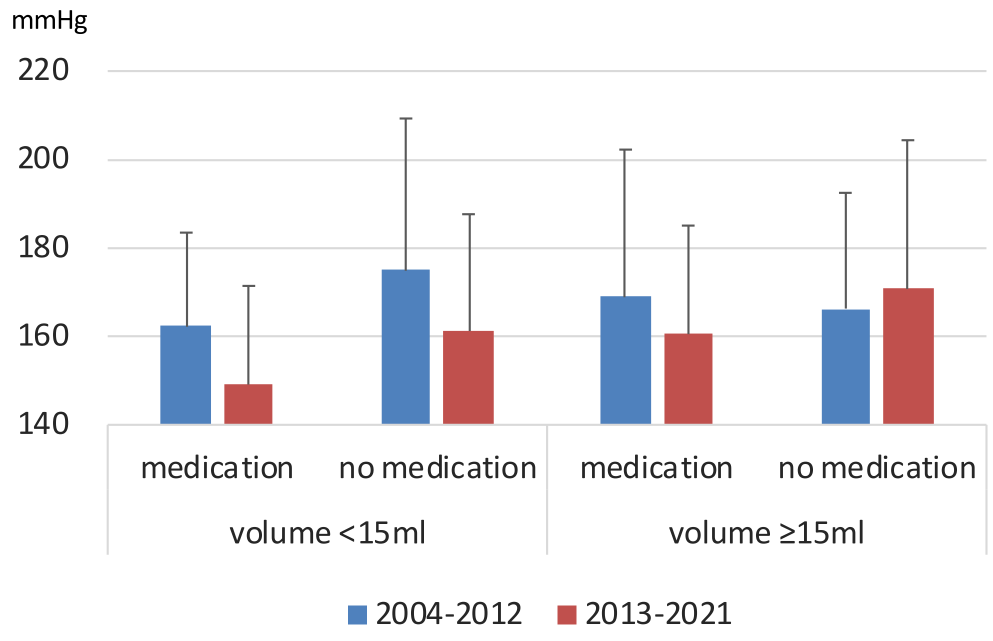

Supplementary Figure S1. Systolic blood pressure (SBP) values according to the volume in the subcortical hemorrhages and medication.
