# Supplementary material for: Secular trends and features of thalamic hemorrhages compared with other hypertensive intracerebral hemorrhages: an 18-year single-center retrospective assessment
Source: Front Neurol. 2023 Aug 15;14:1205091. doi: 10.3389/fneur.2023.1205091 (PMC10464616; doi:10.3389/fneur.2023.1205091)
Supplement: Supplementary file 2 [file Image_2.pdf]

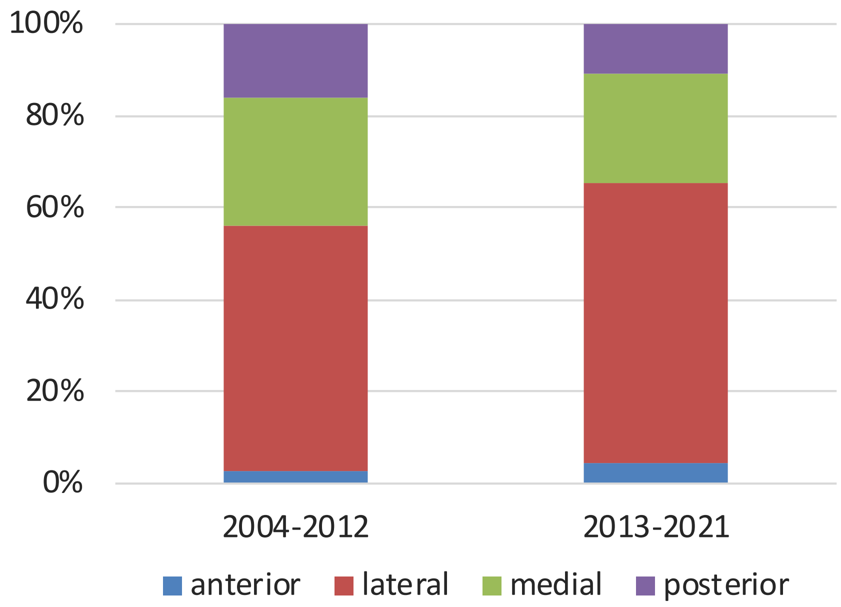

Supplementary Figure S2. The proportions of the specific hematoma locations in the thalamus for each period.
