# Supplementary material for: Secular trends and features of thalamic hemorrhages compared with other hypertensive intracerebral hemorrhages: an 18-year single-center retrospective assessment
Source: Front Neurol. 2023 Aug 15;14:1205091. doi: 10.3389/fneur.2023.1205091 (PMC10464616; doi:10.3389/fneur.2023.1205091)
Supplement: Supplementary file 3 [file Table_1.pdf]

Supplementary Table S1. Difference between the early and late periods for each hypertensive intracerebral hemorrhage site (%)

Hemorrhage site	All	2004–2012	2013–2021	Difference
Thalamus	27.4	24.3	29.3	5.01*
Putamen	27.1	28.9	26.0	−2.87
Subcortex	24.2	22.5	25.2	2.67
Cerebellum	9.2	10.9	8.1	−2.80
Brainstem	8.1	8.1	8.1	−0.02
Caudate	2.5	2.8	2.4	−0.41
Mixed type	1.6	2.5	1.0	−1.58

*$p<0.05$.
